# Characterization of bacterial communities in ticks parasitizing cattle in a touristic location in southwestern China

**DOI:** 10.1007/s10493-023-00799-y

**Published:** 2023-06-07

**Authors:** Yulong Xiang, Jingzhu Zhou, Fuxun Yu, Yan Zhang, Shijun Li, Yong Hu, Wenqin Liang, Qiyong Liu

**Affiliations:** 1 Guizhou Center for Disease Control and Prevention, Guiyang, Guizhou 550004 China; 2 School of Public Health, the key Laboratory of Environmental Polution Monitoring and Disease Control, Ministry of Education, Guizhou Medical University, Guiyang, Guizhou 550025 China; 3 Guizhou Provincial People’s Hospital, Guiyang, Guizhou 550002 China; 4 State Key Laboratory of Infectious Disease Prevention and Control, WHO Collaborating Centre for Vector Surveillance and Management, National Institute for Communicable Disease Control and Prevention, Chinese Center for Disease Control and Prevention, Beijing, 102206 China

**Keywords:** *Rhipicephalus microplus*, *Haemaphysalis longicornis*, Bacterial communities, Guizhou province

## Abstract

The purpose of this study was to investigate tick species around Mount Fanjing and analyze bacterial communities in two species – *Rhipicephalus microplus* and *Haemaphysalis longicornis* – parasitizing cattle in Tongren, Guizhou province, Southwest China, using high-throughput sequencing methods. In April 2019, ticks were collected from five sites in Jiangkou County, Yinjiang County, and Songtao County. In total, 296 ticks were collected, comprising two genera and three species: *H. longicornis*, *Haemaphysalis flava*, and *R. microplus*. *Rhipicephalus microplus* was the most representative species (57.4%) within the collected group, being the dominant species in Tongren City, followed by *H*. *longicornis* (39.5%) and *H. flava* (3.0%). Beta-diversity analysis revealed differences in bacterial community composition among the tick species. The bacterial community structure of *R*. *microplus* collected in the three counties was highly similar. *Chlorella* and *Bacillus* were highly abundant in *H*. *longicornis*. *Rickettsia* was detected at high relative abundance in *R*. *microplus* but in low relative abundance in *H*. *longicornis*, suggesting that *Rickettsia* is more associated with *R*. *microplus* than with *H*. *longicornis*. More in-depth investigations are needed to determine the pathogenic risk of *Rickettsia* and its relationship with the host. This is the first survey on tick-borne bacterial communities in this area, which is of great significance for the prevention and control of tick-borne diseases locally.

**Supplementary Information:**

The online version contains supplementary material available at 10.1007/s10493-023-00799-y.

## Introduction

Ticks are hematophagous ectoparasites that parasitize almost all kinds of vertebrates, including humans, livestock, and wild animals (Zhang et al. 2021a; Sonenshine 2018). Ticks are responsible for spreading a variety of pathogens that threaten their hosts, such as viruses, rickettisae, bacteria, fungi, and protozoa (Brites-Neto et al. 2015), being the second most important disease vectors to humans after mosquitoes, and the most important disease vectors to livestock and wild animals (de la Fuente et al. 2008). Over 900 tick species have been described worldwide (Estrada-Peña 2015), and 124 tick species in total have been reported in China (Chen and Yang 2021), among which 110 are hard ticks (Ixodidae) and 14 soft ticks (Argasidae). In China, it has been proposed that approximately 130 animal species from 20 orders are potentially parasitized by ticks. Moreover, as ticks have a wide geographical distribution (Zhang et al. 2019a), tick-borne diseases occur in several locations throughout China (Huang et al. 2020; Ni et al. 2020; Fang et al. 2015; Wu et al. 2013).

Guizhou province is located in the hinterland of Southwest China, with a warm and humid climate conducive to ticks. In total 19 tick species have been reported in the province, which belong to two families and five genera (Zhang et al. 2019b). Tongren, located in the northeastern part of Guizhou province, with typical karst landforms, is divided east and west by the Wuyi Mountains with Mount Fanjing as the main peak. Mount Fanjing is located in national conservation and a 5A-level tourist attraction, being also listed as a world natural heritage. It is a suitable habitat for ticks due to its unique geographical environment and diverse animal and plant resources. Thus, bacteria carried by local ticks may threaten livestock, wild animals, and tourists. However, the geographical distribution of tick species in Guizhou province as well as the diversity of tick-borne pathogens have yet to be well known, with only a few studies reporting the prevalence of tick-borne diseases, such as Lyme disease and Q fever (Wu et al. 2013).

Therefore, the purposes of this study were to (i) investigate the distribution of tick species parasitizing cattle in three counties in the vicinity of Mount Fanjing (i.e., Jiangkou County, Yinjiang County, and Songtao County) and (ii) characterize bacterial communities by *16S rDNA* high-throughput sequencing of the most prevalent tick species collected in the corresponding geographical area. As *R*. *microplus* and *H*. *longicornis* were the main tick species collected in the study period and location, the diversity and composition of bacterial communities in these two tick species were the focus of the present work.

## Materials and methods

### Geographical location and sample collection

Mount Fanjing is located at the junction of the three counties Yinjiang, Jiangkou, and Songtao, between 27°49’50”–28°1’30”N and 108°45’55”–108°48’30”E. In April 2019, five locations were selected in the vicinity of Mount Fanjing for tick collection as there are suitable environments for ticks. Ticks were collected on the body surface of herding cattle with the owner’s consent. They were collected using forceps by first anesthetizing the ticks with 75% ethanol, then holding the capitulum of the ticks with forceps and gently shaking them from side to side until the capitulum came off. Collected ticks were placed in prepared 15-mL cryotubes in liquid nitrogen tanks, sent to the laboratory, and placed at − 80 °C until later use. Distribution of the sample collection sites (Figure S1) was as follows – Jiangkou County: (i) Kaiwen Village, Taiping Town, (ii) Banpo Village, Bapan Town; Yinjiang County: (iii) Heshui Village, Heshui Town; Songtao County: (iv) Dapingcha Village, Shichang Township, (v) Qiandong Caohai, Panshi Town.

### Tick identification

Morphological features of ticks were identified based on taxonomic and morphological criteria (Teng and Jiang 1991; Chen and Yang 2021) using an SZX7 stereomicroscope (Olympus, Chaoyang, Beijing, China) and a Smartzoom 5 digital microscope (Zeiss, Pudong, Shanghai, China). Observed structures included the shape of basis capituli, porose area, palp, scutum, and peritreme, as well as the dentition formula of hypostome, the presence or absence of festoon, among other structures.

### High-throughput sequencing

#### Sample grouping

Based on collection site, tick species, and tick size, representants of the same tick species originating from different collection sites within the same geographical area were pooled. All ticks were fully engorged. The total volume of each sample was approximately the size of a soy bean, and samples were divided into four groups (Table 1) with three replicates each. The negative control used ddH_2_O instead of sample DNA to exclude environmental and reagent contamination, and the rest of the experimental conditions and procedures were consistent with the samples.

**Table 1:** Tick specimen grouping and coding. All ticks were sampled from cattle in Jiangkou (JK), Yinjiang (YJ) and Songtao (ST) counties, in Tongren City, Guizhou province

Sites	Tick species	Sex/life stage	Sample name	No. ticks
i, ii	*Rhipicephalus microplus*	Adult male and female	JK-1	3
			JK-2	3
			JK-3	3
iii	*R. microplus*	Adult male and female	YJ-1	3
			YJ-2	3
			YJ-3	3
iv, v	*R. microplus*	Adult male and female	ST-1	5
			ST-2	5
			ST-3	5
	*Haemaphysalis longicornis*	Nymph	ST-4	10
			ST-5	20
			ST-6	10

#### Sample preparation

Each sample was washed 3× with 75% ethanol (Lircon, Dezhou, Shandong province, China), and then once with 1× PBS buffer (Life Technologies, Grand Island, NY, USA), and placed in a 2-mL centrifuge tube to prepare for nucleic acid extraction.

#### DNA extraction and library construction

Samples were ground to powder with stainless steel grinding balls in a Scientz-48 high throughput tissue grinder (Scientz, Ningbo, Zhejiang province, China). Nucleic acid extraction was carried out using E.Z.N.A. Mag-Bind Soil DNA Kit (Omega Bio-Tek, Norcross, GA, USA) following the manufacturer’s instructions, and DNA concentration was quantified in a Qubit v.3.0. fluorometer (Invitrogen, Carlsbad, CA, USA). Primers were universal primers for the V3–V4 hyper-variable region of the bacterial *16S rDNA* gene (341 F: CCTACGGGNGGCWGCAG and 785R: GACTACHVGGGTATCTAATCC) (Klindworth et al. 2013). PCR amplifications were performed as follows: initial denaturation at 94 °C for 3 min; followed by five cycles of denaturation at 94 °C for 30 s, annealing at 45 °C for 20 s, extension at 65 °C for 30 s; then 20 cycles of denaturation at 94 °C for 20 s, annealing at 55 °C for 20 s, and extension at 72 °C for 30 s; and a final extension at 72 °C for 5 min. A second bridge PCR was conducted with Illumina-compatible primers to complete library construction, and PCR amplification conditions were as follows: initial denaturation at 95 °C for 5 min; followed by five cycles of denaturation at 94 °C for 20 s, annealing at 55 °C for 20 s, and extension at 72 °C for 30 s; and a final extension step at 72 °C for 5 min.

#### Library quality control and sequencing

Library quality was determined by electrophoresis on 2% agarose gels. DNA fragments of approximately 400 bp were recovered using magnetic beads. The quality and concentration of prepared libraries were determined using Qubit v.3.0 fluorometer. High-throughput sequencing was performed using the Illumina MiSeq platform by Sangon Biotech (Shanghai). The company also performed a bioinformatic analysis for us.

### Bioinformatics analysis

Raw sequence image data obtained by high-throughput sequencing were analyzed by base calling and transformed into raw sequenced reads, and saved as FASTQ files. Adapters were removed using cutadapt v.1.18, and paired-end reads were merged using PEAR v.0.9.8 using the overlapping method. Finally, quality control and filtering were carried out in each sample to obtain clean data. Operational taxonomic unit (OTU) clustering was performed on clean sequences, and chimera sequences were removed to obtain optimal OTU sequences, which were selected as representative sequences with ≥ 97% similarity. Then the generated OTUs were compared with the ribosomal database project (RDP, http://rdp.cme.msu.edu/index.jsp). Sequence classification assignments were performed using the RDP classifier, which was based on Bergey’s taxonomy, and the Naive Bayesian assignment algorithm was used to calculate the probability value of each sequence assigned to various hierarchical levels.

Six metrics (indices) were used for determining alpha-diversity analysis: Shannon, Chao, ACE, Simpson, Shannoneven, and Coverage. The Shannon and Simpson indices indicate the diversity of bacterial communities in the sample, and the Chao and ACE indices indicate the abundance of bacterial communities. The Shannoneven index was used to reflect the evenness of bacterial communities, and the Coverage index reflects the coverage of each sample library.

Data analysis was performed in the statistical language R (v.3.2). Hierarchical clustering was based on the Bray-Curtis distance algorithm, and the package ‘ape’ (v.5.3) of R was used to construct dendrograms.

## Results

### Tick species identification

In total, 296 ticks were collected from two tick genera and three tick species in the three counties included in the study (Table 2). The ticks were identified morphologically as *R. microplus* (n = 170), *H. longicornis* (n = 117), and *Haemaphysalis flava* (n = 9). The most prevalent tick species was *R*. *microplus* (57.4%), followed by *H*. *longicornis* (39.5%) and *H. flava* (3.0%).

**Table 2:** Number of ticks collected from Jiangkou, Yinjiang and Songtao counties

Species	Site i	Site ii	Site iii	Site iv	Site v	Total
*Rhipicephalus microplus*	9	14	113	22	12	170
*Haemaphysalis longicornis*	0	0	0	46	71	117
*Haemaphysalis flava*	2	7	0	0	0	9
Total	11	21	113	68	83	296

### *16S rDNA* sequencing

**Fig. 1:**
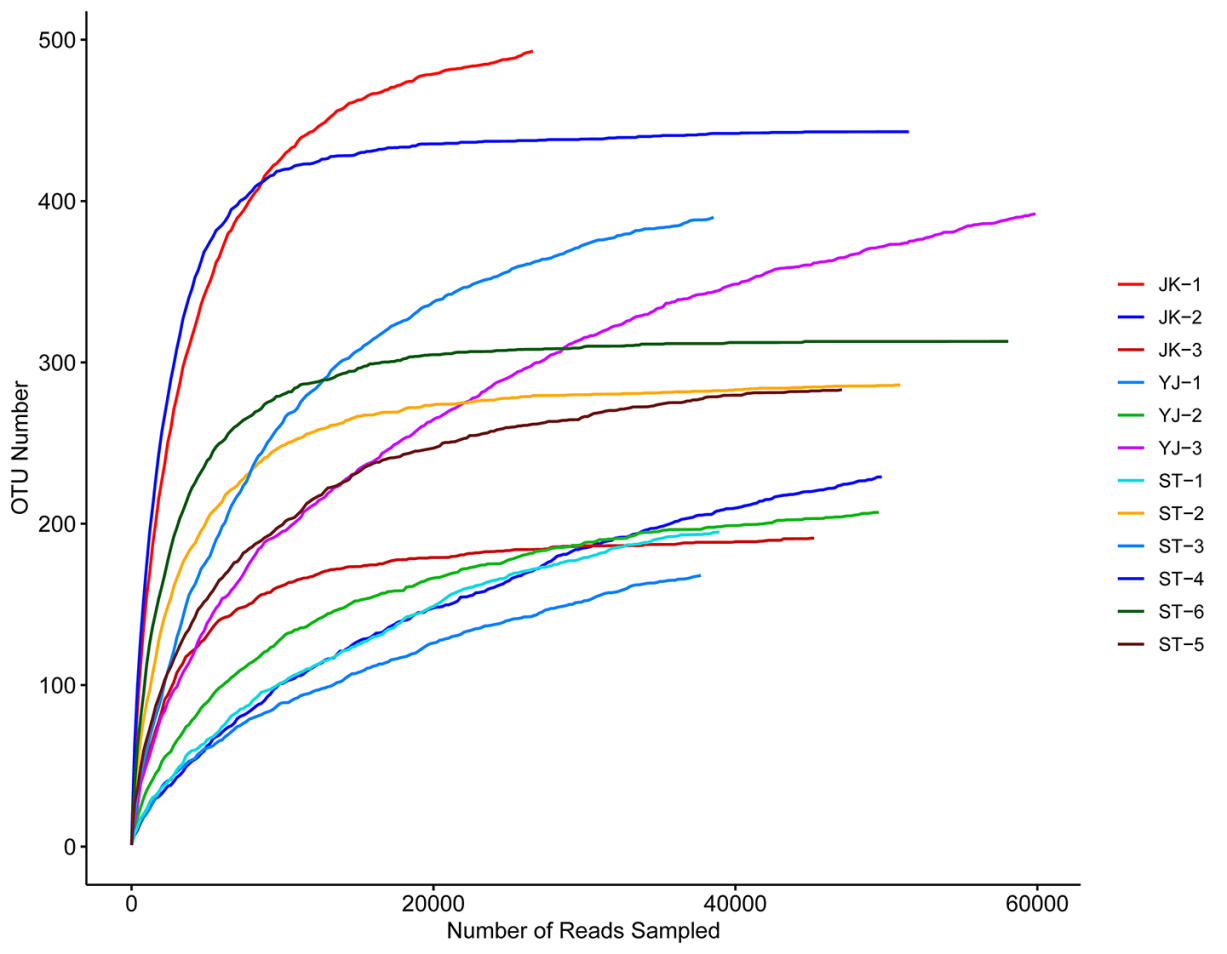
Rarefaction curve showing Operational Taxonomic Unit (OTU) abundance in samples included in the study. See Table 1 for details on the sample codes

The effective number of reads (Table 3) in samples was within the range of 30,000–65,000, and the average length was approximately 400 bp. In total, 904 OTUs were identified in all samples. A rarefaction curve was constructed with the number of reads in the horizontal axis and the number of OTUs in the vertical axis (Fig. 1). The observed number of OTUs increased with sequencing depth, and the curve tended to become flat towards its end, indicating that sequencing depth was satisfactory to cover all bacterial species in the sample. Thus, sequencing data could likely reflect the features of the tick-borne bacterial community in the investigated geographical area.

**Table 3:** Number and length of sequences of each sample in Tongren City, Guizhou province. See Table 1 for details on the sample codes

Sample	Sequence nr.	Base nr.	Mean length (bp)	Minimum length (bp)	Maximum length (bp)
JK-1	31,370	13,030,673	415.39	350	462
JK-2	52,029	21,075,080	405.06	350	468
JK-3	48,290	19,735,317	408.68	352	449
YJ-1	39,708	16,093,527	405.30	351	462
YJ-2	52,064	21,167,178	406.56	355	475
YJ-3	64,724	26,363,800	407.33	351	472
ST-1	41,249	16,705,263	404.99	354	447
ST-2	54,266	22,302,237	410.98	350	468
ST-3	42,751	17,439,659	407.94	350	473
ST-4	57,140	23,924,666	418.70	350	475
ST-5	57,934	24,687,375	426.13	356	433
ST-6	62,550	26,263,073	419.87	351	472

### Analysis of species diversity and structure of bacterial communities in ticks

#### Alpha diversity

Collectively, the obtained data revealed that the diversity and abundance of microbial communities in each sample differed. Diversity was higher in samples JK-1 (*R. microplus*) and ST-4 (*H. longicornis*) and lower in samples JK-2, ST-1, and YJ-1 (all three *R. microplus*). Abundance was highest in sample JK-1 and lowest in sample JK-3 (*R. microplus*). Community distribution in samples JK-1 and ST-4 was more uniform than in the other samples. Coverage indices of all tested samples were > 99.7%, which indicated the reliability of our sequencing data pertaining the microbial community and low probability of sequences not being detected (Table 4).

**Table 4:** Statistics of alpha-diversity index analysis. See Table 1 for details on the sample codes

Sample	OTUs	Shannon	Chao	Ace	Simpson	Shannoneven	Coverage
JK-1	493	2.984	505.000	502.916	0.187	0.481	0.999
JK-2	229	0.402	366.500	356.151	0.870	0.074	0.998
JK-3	191	1.206	197.429	193.791	0.566	0.230	1.000
YJ-1	168	0.478	241.182	249.126	0.829	0.093	0.998
YJ-2	207	0.750	222.037	220.090	0.729	0.141	0.999
YJ-3	392	0.891	462.443	475.789	0.680	0.149	0.998
ST-1	195	0.438	236.143	250.077	0.871	0.083	0.998
ST-2	286	1.905	295.167	289.027	0.359	0.337	1.000
ST-3	390	1.180	445.882	432.888	0.609	0.198	0.998
ST-4	443	2.969	443.857	443.970	0.298	0.487	1.000
ST-5	283	2.024	292.857	291.451	0.293	0.359	0.999
ST-6	313	1.928	313.000	313.158	0.502	0.336	1.000

OTUs, represents the number of operational taxonomic units obtained by OTU clustering

#### Bacterial community composition

**Fig. 2:**
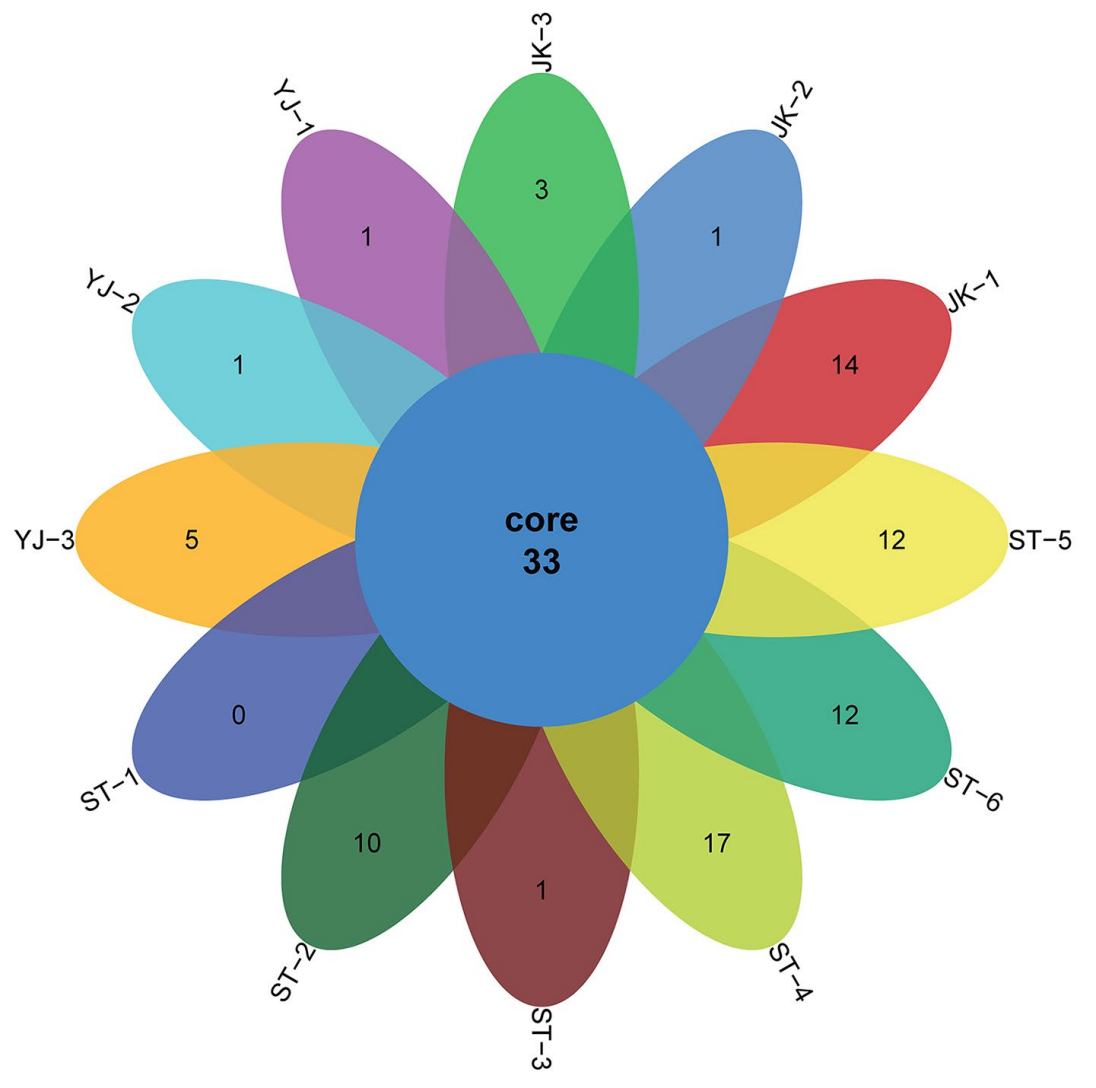
Venn diagram depicting the frequency of microorganisms identified at genus level in ticks collected in the study. The number depicted in the core indicates the frequency of species commonly present in all samples. See Table 1 for details on the codes in the periphery

**Fig. 3:**
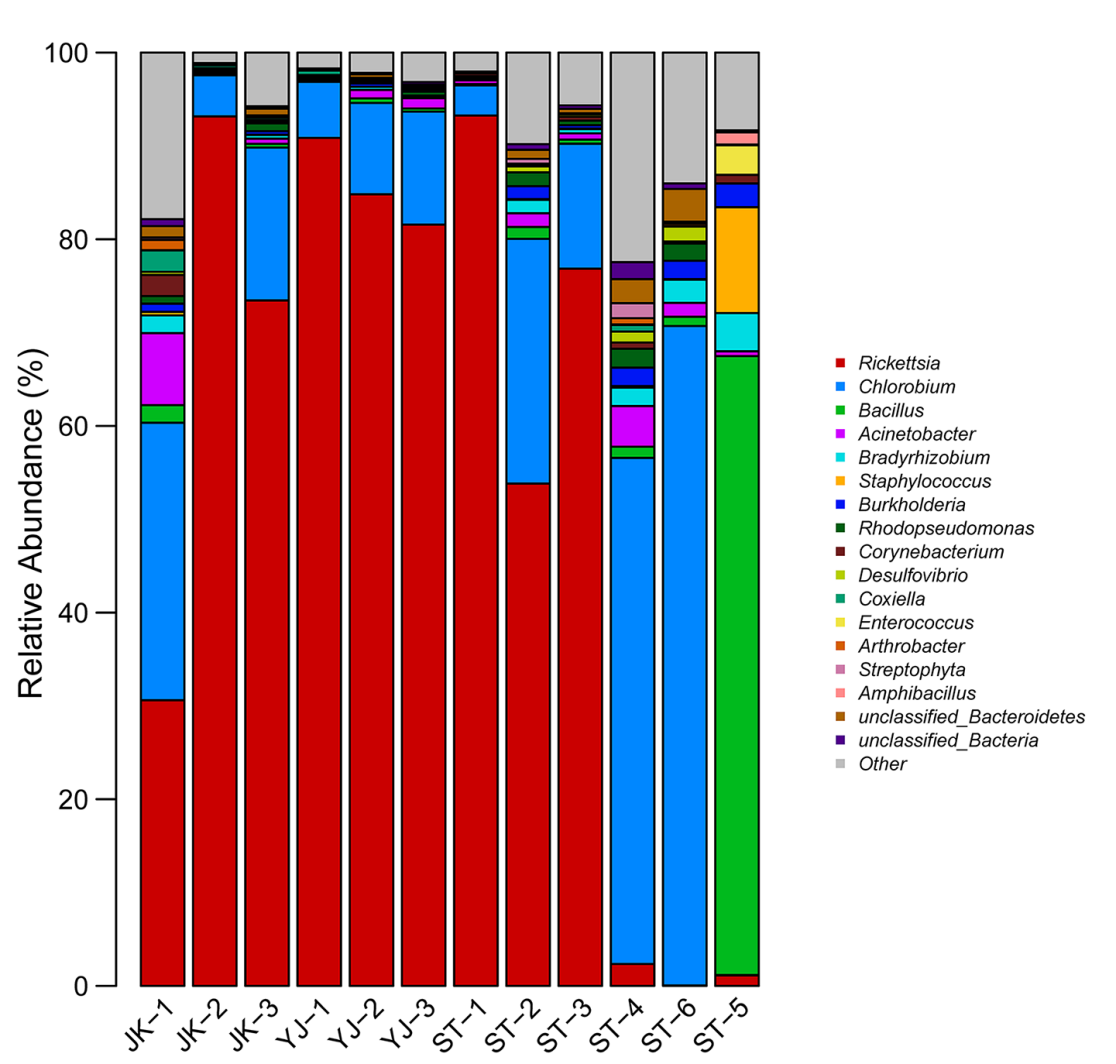
Relative abundance of microbial genera in ticks. Genera whose relative abundance was < 1% in all samples were depicted as ‘other’. See Table 1 for details on the sample codes

**Fig. 4:**
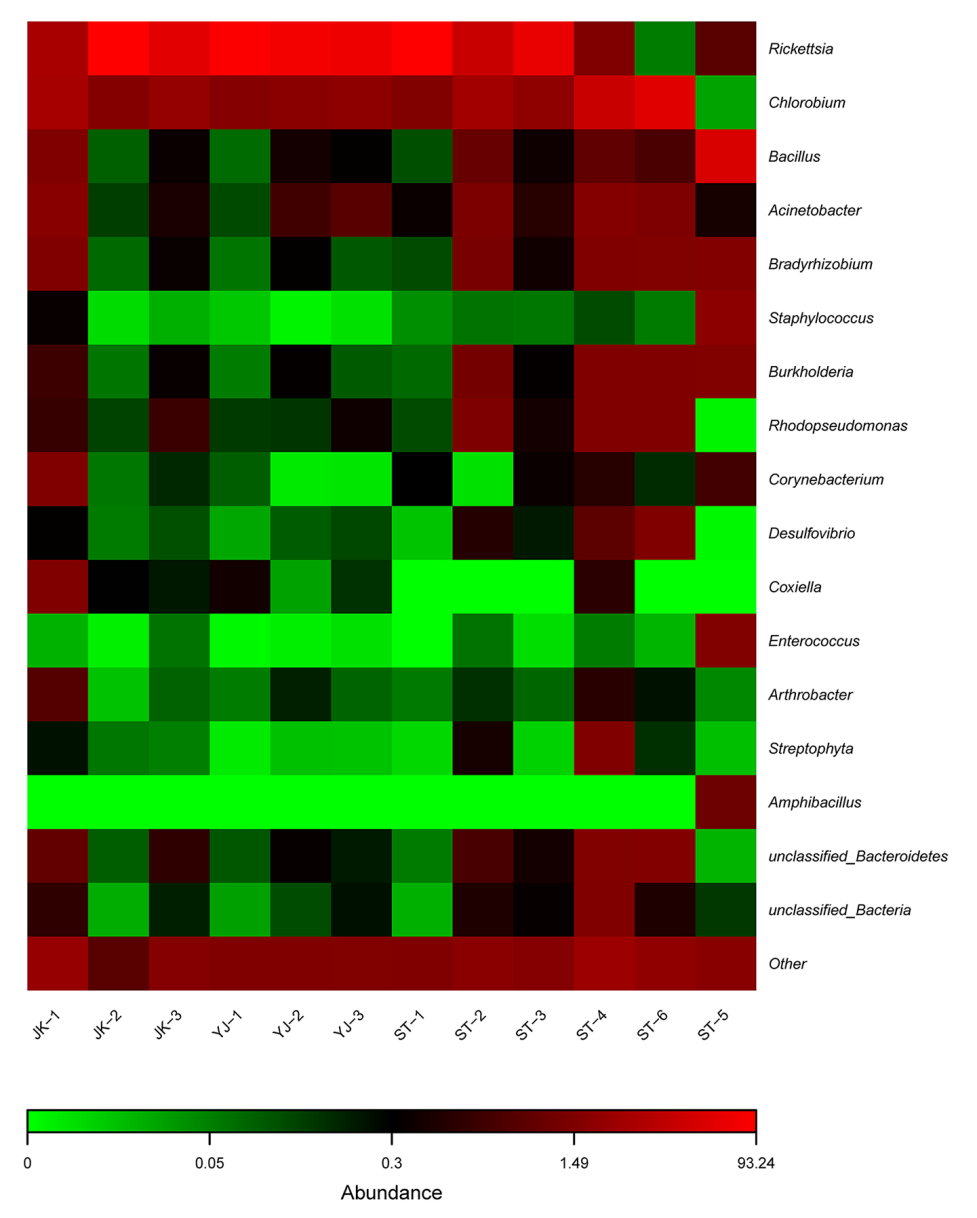
Heat map of the relative abundance of microorganisms identified at genus level in tick samples evaluated in the study. See Table 1 for details on the sample codes

In total, 110 genera, 70 families, 40 orders, 28 classes, and 14 phyla were identified in the 12 samples analyzed. The Venn diagram (with data corresponding to genus level taxa) showed that 33 genera, 42 families, 25 orders, 18 classes, and 12 phyla were shared among the sample groups (Fig. 2). The stacked bar graph and the heat map present the relative abundance of bacterial species in all sample groups, showing only species whose abundance was above 1%. At phylum level, Proteobacteria (65.0%) were the most abundant, followed by Chlorobi (20.5%) and Firmicutes (9.7%); Proteobacteria was the most abundant phylum (> 50%) in nine samples of *R*. *microplus*, whereas Chlorobi was the most abundant (> 50%) in two samples of *H*. *longicornis*, and Firmicutes (> 80%) in one sample of *H*. *longicornis*. At class level, Alphaproteobacteria was detected in high abundance (> 50% in eight samples), with the highest abundance found in sample JK-2 (93%) of *R*. *microplus*. At order level, Rickettsiales was detected in all samples, with the highest relative abundance (> 90%) in samples JK-2, YJ-1, and ST-1. At family level, Rickettsiaceae (56.8%), Chlorobiaceae (20.5%), and Bacillaceae (6.1%) were detected at the highest relative abundance on the whole. At genus level (Figs. 3 and 4), *Rickettsia* was detected in high relative abundance in *R*. *microplus* (> 30%), whereas the relative abundance of *Chlorobium* was highest in samples ST-4 (54.2%) and ST-6 (70.7%), that of *Bacillus* was highest in sample ST-5 (66.3%). However, the content of *Rickettsia* in *H*. *longicornis* was extremely low (< 2.4%), and in the remaining samples, *Bacillus* was present at approximately 1%. The top-10 genera, ranked according to their average relative abundance, are *Rickettsia* (56.8%), *Chlorobium* (20.5%), *Bacillus* (6.1%), *Acinetobacter* (1.6%), *Bradyrhizobium* (1.1%), *Staphylococcus* (1.0%), *Burkholderia* (0.84%), *Rhodopseudomonas* (0.72%), *Corynebacterium* (0.43%), and *Desulfovibrio* (0.38%). As a common endosymbiont of ticks, *Coxiella* was found in low abundance in seven samples. Only sample JK-1 has a relative abundance of 2.3%, and the rest of samples are < 1% (Table S1).

#### Beta diversity

**Fig. 5:**
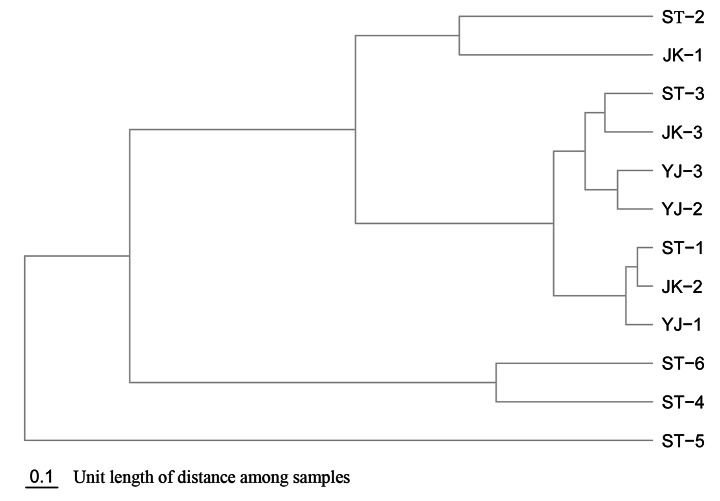
Cluster analysis of tick samples evaluated in the study. See Table 1 for details on the sample codes

Beta-diversity analysis was conducted to determine the dissimilarity of bacterial communities in ticks evaluated in the study. Two main branches (Fig. 5) were identified in dendrograms: *R. microplus* samples were found in a branch regardless of the site of collection, and *H. longicornis* samples fell in other branches. Sample ST-5 differed from the other 11 samples in the corresponding branch due to the presence of *Bacillus* (66.3%), *Staphylococcus* (11.4%), and very little *Chlorobium* (0.04%); sample ST-4 was similar to sample ST-6 as the abundance of *Chlorobium* was 50% and *Rickettsia* was found in low abundance in both samples, 2.4 and 0.05%, respectively.

## Discussion

The geographical distribution of the collected ticks was different: in sites i and ii (in Jiangkou County), the tick species were highly similar (mainly *R*. *microplus*); in site iii (Yinjiang County), *R*. *microplus* was the only species identified, and in sites iv and v (Songtao County) *H*. *longicornis* was the dominant species. The environment of the five sampling sites is similar and all locations are near Mount Fanjing, and they all have a subtropical humid monsoon climate. The average annual temperature is 16–17 ℃, the average temperature of the hottest month (July) is about 27 ℃, and that of the coldest month (January) is about 5 ℃. But sampling site v has some uniqueness: it has 230 ha of artificial pasture, stocked with cattle, sheep, rabbits, and other animals; the other sampling sites only stocked cattle. Perhaps this explains the difference in the dominant tick species. *Rhipicephalus microplus* is the main ectoparasite affecting livestock worldwide (Silva et al. 2016), whereas *H. longicornis* is a tick species widely distributed in China (Malik et al. 2019).

In this study, using *16S rDNA* sequencing analysis to explore the diversity of bacterial communities in collected ticks, the species *Rickettsia*, *Chlorobium*, and *Bacillus* were detected in all 12 samples. *Rickettsia* was detected at the highest relative abundance in *R*. *microplus*. Lim (2020) applied high-throughput sequencing to characterize the diversity of bacterial communities in ticks and also detected *Rickettsia* in high abundance in *Dermacentor atrosignatus* and *Dermacentor compactus*. Bacteria within the genus *Rickettsia*, including spotted fever group rickettsiae (SFGR), are mainly transmitted to animals and humans by tick bites; the genus *Rickettsia* has at least 30 species distributed worldwide, of which 21 are considered pathogens (Satjanadumrong et al. 2019). SFGR are known to cause a variety of natural focal diseases, including the Rocky Mountain spotted fever (RMSF) (Zazueta et al. 2021) in the Americas and the Mediterranean spotted fever in parts of Asia, Europe, and Africa (Satjanadumrong et al. 2019). In Europe, SFGR is chiefly transmitted by *Dermacentor* (Buczek et al. 2020), whereas in Asia, *Dermacentor* and *Haemophysalis* are most frequently associated with rickettsial carriage. SFGR is highly prevalent in northern China. Ten validated SFGR species have been discovered in ticks and vertebrate hosts (Wang et al. 2021). Nine samples (75%) evaluated in this study were shown to contain a high relative abundance of *Rickettsia* – they may be pathogens or endosymbionts, so we need further investigation to assess the possible risk posed by *R. microplus*.

In addition, seven samples were shown to contain a low abundance of *Coxiella*, ticks collected in Jiangkou County (samples JK-1, 2, and 3), Yinjiang County (YJ-1, 2, and 3), and Songtao County (ST-4). These findings differed from those of Guizzo et al. (2017), in which *Coxiella* accounted for 99% of the bacterial community in *R*. *microplus* eggs and 98.3% in *R*. *microplus* larvae. These differences may be attributed to the stage of development of ticks and the surveyed geographical location, but of course this requires confirmation. Bacteria within the genus *Coxiella* establish a symbiotic relationship with ticks and are able to infect ticks at all stages of their lifecycle (Ni et al. 2020). *Coxiella burnetii* is an obligate intracellular bacterium and the agent of Q fever in humans; Q fever is a zoonotic disease widely prevalent worldwide (except for New Zealand), characterized by high infectivity and long-term environmental persistence (Long et al. 2019; Klemmer et al. 2018). Ticks were not considered vectors of *C. burnetii* previously (Abdelkadir et al. 2019), but the results of a recent study support the link between ticks and Q fever, with the latter 3× more likely to occur where the former is found (Hussain et al. 2022). Ticks play an important role in the wild and peridomestic cycles of *C. burnetii* worldwide, having been isolated from at least 40 tick species within the Ixodidae and 14 tick species of the Argasidae (Bolaños-Rivero et al. 2017). Although animals infected with *C. burnetii* are often asymptomatic, sheep, goats, and cattle may experience abortion, premature birth, stillbirth, and weak offspring (Di Domenico et al. 2014), thus the bacterium can disseminate in the environment through birth products as well as the urine and milk of infected animals (Rodolakis et al. 2007). Moreover, as it is extremely resistant to desiccation and radiation, *C. burnetti* can persist in soil and other dry surfaces for long periods (Körner et al. 2021). Humans are very sensitive to *C. burnetii*, especially those who work with livestock and are exposed to birth products, infectious dust particles, contaminated wool, and highly infectious aerosols. Infections by *C. burnetii* are often occupation-related, being highly prevalent among slaughterhouse workers, livestock handlers, veterinarians, and farmers (Frangoulidis et al. 2021; Klemmer et al. 2018; Esmaeili et al. 2014).

In the present study, Proteobacteria were detected at the highest abundance in *R*. *microplus*, whereas Chlorobi and Firmicutes were at the highest abundance in *H*. *longicornis*, thus indicating that bacterial communities differ among tick species. *Chlorobium* was detected in ticks evaluated in the current study, which are bacteria that mainly live in water and are able to perform photosynthesis; the presence of this bacterium in *H*. *longicornis* may be a result of tick’s water ingestion habits. For instance, *Amblyomma americanum* and *Ixodes scapularis* have been shown to be able to actively ingest liquid water from the environment to compensate for water loss occurring due to excretion processes (Maldonado-Ruiz et al. 2020, 2021; Kim et al. 2017, 2019). Therefore, *Chlorobium* was likely ingested along with water by *H*. *longicornis*. Of course, the possibility that these bacteria originated from the surface of the exoskeleton and the host skin could not be ruled out, as it is quite difficult to completely remove surface contaminants from the tick exoskeleton.

Interestingly, the results of the present study differed from previous studies conducted in other provinces which employed high-throughput sequencing technology. Zhang et al. (2019c) evaluated the bacterial communities of *H*. *longicornis,* in which *Coxiella* was shown to be the dominant genus, and *Rickettsia* was not detected. Xiang et al. (2017) studied the bacterial communities of saliva obtained from engorged adult *R. microplus* females in Hunan province and found that Proteobacteria was the dominant phylum, and *Acinetobacter*, *Rickettsia*, *Escherichia*, and *Coxiella* were the major genera. Zhang et al. (2021b) also detected a high abundance of *Rickettsia* in *H. longicornis* in Shandong province. At present, it is not possible to provide a comprehensive explanation of the changes in bacterial richness observed in the current study with those described in previous studies. Multiple factors can determine changes in tick microbiome, including tick species, sample collection site, presence of host blood, and degree of engorgement (Clow et al. 2018; Estrada-Peña et al. 2018; Swei and Kwan 2017; Van Treuren et al. 2015; Moreno et al. 2006). In addition, several bacterial species detected in the study, including *Acinetobacter* and *Staphylococcus*, are commonly detected in ticks, indicating that these bacteria may play a biological role in these hosts. Moreover, it is worth noting that species from the genera *Rickettsia* and *Coxiella* are common tick endosymbionts (Dall’Agnol et al. 2021; Lim et al. 2020; Maldonado-Ruiz et al. 2020). Most importantly, the high carrying rate of *Rickettsia* in ticks evaluated in the study sites towards the need to strengthen future investigations to identify vector competence and potential epidemiological risk of tick-borne disease in the surveyed area.

Finally, high-throughput *16S rDNA* sequencing has become the standard method for microbial classification and identification, which enables the identification of bacteria found in very low abundance and/or non-cultivable states. However, this method has certain limitations, such as accuracy and comprehensiveness being associated with the reference database (Couper and Swei 2018). Considering only the V3–V4 hypervariable regions of the *16S rDNA* gene, sequence length was likely insufficient to enable the identification of bacteria at the species level. *16S rDNA* full-length sequencing technology yields sequences of approximately 1542 bp in length, including nine hypervariable regions and 10 conserved regions, which would allow for further classification and identification of bacterial communities at the species level (Dong et al. 2021). Unfortunately, the limited number of samples did not allow further investigation of the identity of these bacteria to establish whether these bacteria are pathogens or not. However, the high-throughput sequencing method employed in the present study enabled the successful exploration of the bacterial communities carried by ticks parasitizing cattle in Tongren, Guizhou province, and highlighted variation in bacterial communities found in *R*. *microplus* vs. *H*. *longicornis*, the most prevalent tick species in the surveyed areas. Collectively, the results discussed herein provide a scientific basis for strengthening prevention and control measures of ticks and tick-borne diseases in Guizhou province.

## Conclusions

The present study constitutes a survey of tick distribution affecting cattle in Tongren, Guizhou province, China, as well as of the diversity and composition of microbial communities found in the two tick species most prevalent locally. Regional differences were found in the distribution of tick species as well as in bacterial species carried by these parasites. Future studies should comprise an in-depth analysis of tick microbiota composition at the species level and explore the role of the identified microorganisms in ticks.

## Electronic supplementary material

Supplementary material

## Data Availability

The datasets generated during and analysed during the current study are available from the corresponding author on reasonable request.
