# Supplementary material for: Characterization of bacterial communities in ticks parasitizing cattle in a touristic location in southwestern China
Source: Exp Appl Acarol. 2023 Jun 7;90(1-2):119–35. doi: 10.1007/s10493-023-00799-y (PMC10293413; doi:10.1007/s10493-023-00799-y)
Supplement: Supplementary file 1 — Supplementary material [file 10493_2023_799_MOESM1_ESM.docx]

**Supplementary Information**

**Fig. S1** Tick sampling sites

**Table S1** Relative abundance and average relative abundance of bacteria at the genus level among samples

|  | JK-1 | JK-2 | JK-3 | YJ-1 | YJ-2 | YJ-3 | ST-1 | ST-2 | ST-3 | ST-4 | ST-6 | ST-5 | average  relative  abundance (%) |  |  |
| --- | --- | --- | --- | --- | --- | --- | --- | --- | --- | --- | --- | --- | --- | --- | --- |
| Rickettsia | 30.60311 | 93.14765 | 73.44158 | 90.84664 | 84.80703 | 81.56506 | 93.24376 | 53.8182 | 76.84664 | 2.352233 | 0.053391 | 1.134818 | 56.82 |  |  |
| Chlorobium | 29.73776 | 4.423336 | 16.36508 | 6.018248 | 9.789857 | 12.09955 | 3.229121 | 26.22461 | 13.37986 | 54.2179 | 70.64689 | 0.036127 | 20.51 |  |  |
| Bacillus | 1.888709 | 0.112696 | 0.402601 | 0.090181 | 0.488988 | 0.335727 | 0.146428 | 1.266842 | 0.44366 | 1.200396 | 0.985154 | 66.2976 | 6.14 |  |  |
| Acinetobacter | 7.701569 | 0.177094 | 0.553024 | 0.153838 | 0.903213 | 1.117421 | 0.40075 | 1.477 | 0.661599 | 4.360663 | 1.488064 | 0.516406 | 1.63 |  |  |
| Bradyrhizobium | 1.903759 | 0.094585 | 0.393753 | 0.068962 | 0.335421 | 0.123601 | 0.151566 | 1.422006 | 0.464416 | 1.963755 | 2.492164 | 4.095121 | 1.13 |  |  |
| Staphylococcus | 0.376237 | 0.014087 | 0.030969 | 0.021219 | 0.004041 | 0.011692 | 0.043671 | 0.074636 | 0.067457 | 0.153449 | 0.058558 | 11.35881 | 1.02 |  |  |
| Burkholderia | 0.876632 | 0.068423 | 0.373844 | 0.0557 | 0.347545 | 0.121931 | 0.09505 | 1.384688 | 0.33988 | 1.981237 | 1.966863 | 2.522526 | 0.84 |  |  |
| Rhodopseudomonas | 0.816434 | 0.167032 | 0.842808 | 0.185667 | 0.193979 | 0.437615 | 0.151566 | 1.480929 | 0.500739 | 2.025911 | 1.846302 | 0.00425 | 0.72 |  |  |
| Corynebacterium | 2.268708 | 0.064398 | 0.22121 | 0.114052 | 0.008082 | 0.010022 | 0.297994 | 0.011785 | 0.404743 | 0.670124 | 0.215287 | 0.92018 | 0.43 |  |  |
| Desulfovibrio | 0.331088 | 0.058361 | 0.141574 | 0.034481 | 0.119216 | 0.158677 | 0.02312 | 0.644224 | 0.251667 | 1.177088 | 1.615514 | 0.002125 | 0.38 |  |  |
| Coxiella | 2.291283 | 0.295828 | 0.252179 | 0.477428 | 0.036371 | 0.202105 | 0 | 0 | 0 | 0.70703 | 0 | 0 | 0.36 |  |  |
| Enterococcus | 0.030099 | 0.006037 | 0.075211 | 0.002652 | 0.006062 | 0.011692 | 0 | 0.074636 | 0.012973 | 0.054387 | 0.029279 | 3.17069 | 0.29 |  |  |
| Arthrobacter | 1.102374 | 0.024149 | 0.106181 | 0.053048 | 0.236411 | 0.101887 | 0.061654 | 0.208194 | 0.095997 | 0.677894 | 0.265234 | 0.046753 | 0.25 |  |  |
| Streptophyta | 0.267128 | 0.06641 | 0.050878 | 0.007957 | 0.024247 | 0.023384 | 0.015413 | 0.510665 | 0.018162 | 1.596644 | 0.208398 | 0.025502 | 0.23 |  |  |
| Amphibacillus | 0 | 0 | 0 | 0 | 0 | 0 | 0 | 0 | 0 | 0 | 0 | 1.313329 | 0.11 |  |  |
| unclassified_Bacteroidetes | 1.215245 | 0.114709 | 0.741052 | 0.129967 | 0.369772 | 0.248873 | 0.059085 | 0.97812 | 0.485172 | 2.581435 | 3.503152 | 0.029752 | 0.87 |  |  |
| unclassified_Bacteria | 0.741187 | 0.032199 | 0.241118 | 0.037133 | 0.149525 | 0.268916 | 0.030827 | 0.599049 | 0.363231 | 1.827788 | 0.597637 | 0.189136 | 0.42 |  |  |
| Other | 17.84868 | 1.133002 | 5.766934 | 1.702827 | 2.180238 | 3.161851 | 2.049991 | 9.82441 | 5.663804 | 22.45207 | 14.02811 | 8.336875 | 7.85 |  |  |
